# Measures of Light in Studies on Light-Driven Plant Plasticity in Artificial Environments

**DOI:** 10.3389/fpls.2012.00156

**Published:** 2012-07-17

**Authors:** Ülo Niinemets, Trevor F. Keenan

**Affiliations:** ^1^Institute of Agricultural and Environmental Sciences, Estonian University of Life SciencesTartu, Estonia; ^2^Department of Organismic and Evolutionary Biology, Harvard UniversityCambridge, MA, USA

**Keywords:** dry mass per unit area, greenhouse transmittance, growth chambers, lighting in plant growth, meta-analysis, nitrogen content, photosynthetic capacity, standardization of environmental conditions

## Abstract

Within-canopy variation in light results in profound canopy profiles in foliage structural, chemical, and physiological traits. Studies on within-canopy variations in key foliage traits are often conducted in artificial environments, including growth chambers with only artificial light, and greenhouses with and without supplemental light. Canopy patterns in these systems are considered to be representative to outdoor conditions, but in experiments with artificial and supplemental lighting, the intensity of artificial light strongly deceases with the distance from the light source, and natural light intensity in greenhouses is less than outdoors due to limited transmittance of enclosure walls. The implications of such changes in radiation conditions on canopy patterns of foliage traits have not yet been analyzed. We developed model-based methods for retrospective estimation of distance vs. light intensity relationships, for separation of the share of artificial and natural light in experiments with combined light and for estimation of average enclosure transmittance, and estimated daily integrated light at the time of sampling (*Q*_int,C_), at foliage formation (*Q*_int,G_), and during foliage lifetime (*Q*_int,av_). The implications of artificial light environments were analyzed for altogether 25 studies providing information on within-canopy gradients of key foliage traits for 70 species × treatment combinations. Across the studies with artificial light, *Q*_int,G_ for leaves formed at different heights in the canopy varied from 1.8- to 6.4-fold due to changing the distance between light source and growing plants. In experiments with combined lighting, the share of natural light at the top of the plants varied threefold, and the share of natural light strongly increased with increasing depth in the canopy. Foliage nitrogen content was most strongly associated with *Q*_int,G_, but photosynthetic capacity with *Q*_int,C_, emphasizing the importance of explicit description of light environment during foliage lifetime. The reported and estimated transmittances of enclosures varied between 0.27 and 0.85, and lack of consideration of the reduction of light compared with outdoor conditions resulted in major underestimation of foliage plasticity to light. The study emphasizes that plant trait vs. light relationships in artificial systems are not directly comparable to natural environments unless modifications in lighting conditions in artificial environments are taken into account.

## Introduction

Light is a key environmental factor altering plant form and function (Givnish, 1988; Valladares, 2003; Niinemets, 2007; Pearcy, 2007; Poorter et al., 2009a). There are extensive light gradients within-plant canopies and numerous studies have demonstrated that foliage structural, chemical, and physiological traits acclimate to these gradients (e.g., Hirose and Werger, 1987; Gutschick and Wiegel, 1988; Terashima and Hikosaka, 1995; Anten, 2005; Hikosaka, 2005; Niinemets, 2007), resulting in enhanced canopy carbon gain compared with invariable foliage characteristics (e.g., Niinemets and Anten, 2009; Dewar et al., 2012; Niinemets, 2012).

Several classical studies investigating plant acclimation along light gradients have been conducted in artificial environments including greenhouses with natural illumination, greenhouses with natural, and supplemental illumination and growth chambers (e.g., Gutschick and Wiegel, 1988; Hirose et al., 1988; Evans, 1993a,b; Pons et al., 1993). Artificial environments are currently also extensively used for within-plant acclimation studies (e.g., Dreccer et al., 2000; Lötscher et al., 2003; Boonman et al., 2009; Pettersen et al., 2010; Trouwborst et al., 2010). While artificial environments provide a means to investigate plant responses to light without interfering interactions with other environmental drivers (e.g., Niinemets, 2010; Poorter et al., 2012), there are a number of important and often neglected differences in within-canopy light environments between artificial systems and outdoor conditions.

In natural environments, instantaneous values of quantum flux density, *Q*, are strongly variable during the day, between the days and seasons. Thus, average integrated quantum flux density (*Q*_int_) at a specific canopy location or relative quantum flux density (*R*_Q_), the ratio of *Q* at a specific canopy position to *Q* above the canopy (*Q*_0_), are used as estimates of long-term light conditions (Anten, 1997; Meir et al., 2002; Aranda et al., 2004; Niinemets, 2007; Posada et al., 2009; Hallik et al., 2012). As *R*_Q_ can be estimated separately from instantaneous measurements of light profiles (e.g., Parent and Messier, 1996) or by other techniques such as hemispheric photography (e.g., Rich et al., 1993), *Q*_int_ for a specific location in the canopy can be estimated from *R*_Q_ and long-term above canopy estimates of *Q*_0_ (*Q*_int,0_) as:

(1) $$Q_{\text{int}}=R_{\text{Q}}Q_{\mathrm{int},0}$$

This equation is widely used in studies on within-canopy variation in plant traits (Chazdon and Field, 1987; Kull and Tulva, 2002; Meir et al., 2002; Fleck et al., 2003; Niinemets, 2007).

Compared with natural illumination, plant lighting conditions are dramatically different when grown under artificial light. In the case of artificial light, light intensity strongly increases with decreasing distance between the lamp and plant leaves (Gates, 1980; Poorter et al., 2012). Thus, *Q*_int,0_ for topmost leaves varies as the plants increase in size, unless the distance between plants and light source is maintained constant during the experiment. However, when the lamps are raised to maintain the intensity constant at the top of the canopy, light at the bottom of the canopy is inevitably reduced. In contrast to natural canopies where light intensity decreases from canopy top to bottom only due to plant foliage, in experiments with artificial light, light gradients also result from a distance-dependent reduction of light. As a result, light gradients during foliage development and in mature canopies are expected to be stronger than in plants exposed only to natural illumination, and this may affect plant acclimation and relationships with integrated light at the time of foliage sampling. So far, the implications of distance-dependent variations in *Q*_int,0_ on gradients of foliage structural, chemical, and physiological traits have not been investigated, and studies under artificial illumination have been considered to be representative models of field conditions.

The situation is even more complicated in studies using natural illumination supplemented by artificial light as can often occur in experiments conducted in high latitudes in greenhouses beyond the normal growing period when days are short (e.g., Dreccer et al., 2000; Pettersen et al., 2010; Trouwborst et al., 2010). In experiments with combined lighting, incident integrated leaf light is the sum of integrated irradiance from artificial light source that varies with the distance between the foliage and artificial light source, and natural integrated light that fluctuates between the days and varies during the season. The overall effect of distance from the artificial light source strongly depends on the share of total integrated light between artificial and natural light at the top of the canopy, and the degree of distance-dependent reduction of artificial light. Importantly, this share is expected to change as plants increase in size, and consequently, prediction of dynamic changes of light within the canopy illuminated by artificial and natural light sources requires determination of the contributions of natural and artificial light at different vegetation layers.

As noted above, many acclimation studies have examined foliage traits in relation to *R*_Q_, rather than in relation to *Q*_int_. Outdoors, *R*_Q_ = 1.0 typically refers to a completely open location (so-called “full sun”) and *R*_Q_ = 0.0 to an hypothetical situation with no light penetrating at all. Outdoors, a certain maximum value of *Q*_int_, *Q*_int,0_, corresponds to *R*_Q_ = 1.0. However, in studies conduced in artificial environments, *R*_Q_ is commonly taken as 1.0 at the top of the vegetation inside the enclosure (apparent relative light, *R*_Q,A_), implying that *Q*_int_ values different from *Q*_int,0_ can correspond to *R*_Q,A_ = 1.0 inside the enclosure, and generally *R*_Q_ ≠ *R*_Q,A_. As foliage traits adjust to integrated rather than to relative light, this is problematic. In studies with artificial light, variations in *Q*_int_ corresponding to *R*_Q,A_ = 1.0 can vary due to differences in intensity of artificial light used in different studies and variations in overall vegetation height (distance between light source and plants). In studies with natural light, *Q*_int_ for *R*_Q,A_ = 1.0 can vary due to geographical location, season, and weather conditions during the period of interest, whereas in studies with combined supply of natural and artificial light, all the aforementioned factors can result in variations in *Q*_int_ at the top of the canopy.

In the case of enclosures such as greenhouses or microcosms relying on natural or natural and supplemental light, it is also important to consider that the enclosure surface only partly transmits the natural light incident to the enclosure surface (Kittas et al., 1999; Papadakis et al., 2000). Accordingly, *Q*_int_ corresponding to *R*_Q,A_ = 1.0 inside the enclosure also varies due to variations in enclosure transmittance, and is essentially always less than *Q*_int_ above the enclosure. The circumstance that light intensity inside the greenhouse is generally less than outside is not always considered when summarizing the results of past studies by meta-analyses (e.g., Poorter et al., 2009a, 2011). However, typical daily average values of greenhouse transmittances are on the order of 0.4–0.6 (Kittas et al., 1999; Papadakis et al., 2000), implying that consideration of light reduction due to greenhouse material is important to be able to estimate the actual light levels corresponding to *R*_Q,A_ = 1.0 in given studies. The major implication of *R*_Q_ ≠ *R*_Q,A_, is that the parameters of the regressions of foliage, physiological, chemical, and structural traits on *R*_Q,A_, characterizing foliage responsiveness to light, are not comparable with studies in field environments, and furthermore, they are also not comparable among different studies using different lighting setups.

In the current study, we first developed approaches for retrospective estimation of *Q*_int_ values corresponding to *R*_Q,A_ = 1.0 at the top of the canopy for various experimental setups in artificial environments, and methods for the determination of integrated light throughout plant development in artificial systems. Thereafter we tested the hypothesis that foliage structural, chemical, and physiological traits in fast-growing herb canopies are more strongly associated with *Q*_int_ during plant growth than with *Q*_int_ in the fully developed canopies. Finally, we evaluated the implications of *R*_Q_ ≠ *R*_Q,A_ on statistical relationships of foliage key traits on *R*_Q,A_. The results underscore the importance of detailed description of light in studies in artificial environment, and also emphasize that caution should be exercised in interpreting plasticity data obtained in such systems.

## Theory: Light in Manipulated Environments (Microcosms, Greenhouses, Growth Chambers)

### Enclosures with natural lighting

In an enclosure exposed to natural light, the amount of light incident to vegetation (*Q*_en_) is related to the light incident above the enclosure, *Q*_ab_, as:

(2) $$Q_{\text{en}}=\kappa_{\text{Q}}Q_{\text{ab}}$$

where κ_Q_ is the fraction of light penetrating the enclosure (transmittance). κ_Q_ depends on enclosure material optical properties and the angle of incidence of solar radiation. Thus, as the instantaneous values of *Q*_ab_ vary, instantaneous values of κ_Q_ can strongly vary too. In practice, an average value during the study period, κ_Q,av_, is needed to convert the integrated light outside the enclosure (*Q*_int,ab_) to integrated light inside the enclosure (*Q*_int,en_), and to relate the observed (apparent) relative light inside the enclosure, *R*_Q,A_ (*R*_Q,A_ = 1.0 at the top of the vegetation inside the enclosure) to relative light outside the enclosure (*R*_Q_ = 1.0 for a completely open location):

(3) $$R_{\text{Q}}=\kappa_{\text{Q},\text{av}}R_{\text{Q},\text{A}}$$

The transmittance for direct light (κ_Q,dir_) of the cover materials used in greenhouses such as glass and transparent plastic, e.g., plexiglass (acrylic), polyethylene films, etc., decreases with increasing angle of incidence (angle between the light beam incident on a surface and the normal to the surface; Pollet and Pieters, 1999; Altuglas International, 2000). For example, the transmittance of glass for direct beam *Q* is ca. 0.89 for the angle of incidence, ϕ, of 0°, 0.7–0.8 for the angle of incidence of 50°, and only ca. 0.4–0.5 for the angle of incidence of 75° (Pollet and Pieters, 1999, 2002). Thus, transmittance becomes particularly low in morning and evening hours and is generally low in winter months in mid- to high latitudes.

Although the diffuse light transmittance (κ_Q,diff_) of the enclosure materials is less sensitive to the angle of incidence, diffuse transmittance is typically less than the direct transmittance at low angles of incidence (Kittas et al., 1999; Papadakis et al., 2000). For example, diffuse transmittance for glass is 0.72–0.84, for acrylic sheet 0.70, and for low density polyethylene 0.62 (Foster and Stearns, 1971; Pollet and Pieters, 1999; Papadakis et al., 2000). Furthermore, condensation on enclosure surface due to humidity buildup inside the enclosure and surface wetness due to precipitation and dew outside the enclosure can importantly reduce the transmittance. Depending on the angle of incidence, the reduction due to surface water can be 2–10% for glass and 10–25% for various plastic materials (Pollet and Pieters, 2000, 2002). In addition, material aging for various plastic enclosures can alter the material spectral properties and overall light transmittance (Kittas et al., 1999; Papadakis et al., 2000). Thus, the geometry of the enclosure, weather conditions, latitude, and time of the year that alter the angle of incidence for solar radiation importantly affect the enclosure light transmittance. Given the optical properties of the enclosure material for direct and diffuse light, κ_Q,av_ is given as:

(4) $$\kappa_{\text{Q},\text{av}}=\left(1-f_{\text{dif}}\right)\kappa_{\text{Q},\text{dir},\text{av}}+f_{\text{dif}}\;\kappa_{\text{Q},\text{dif}}$$

where κ_Q,dir,av_ is the radiation intensity-weighted and angle of incidence corrected daily average κ_Q,dir_, and *f*_dif_ is the average fraction of diffuse light in total light. *f*_dif_ depends on atmospheric clearness and cloudiness conditions (Misson et al., 2005; Wang et al., 2006; Still et al., 2009).

Although enclosures for plant growth such as greenhouse facilities are typically built outside high density urban areas with tall buildings, we note that apart from the enclosure transmittance, possible structures outside the enclosure can nevertheless alter the light availability relative to full sunlight. Such effects cannot be considered in retrospective analyses, but we argue that modification of light availability by buildings and vegetation outside the enclosure should be assessed in future studies. Reduction of light intensity by outside structures can be assessed by simultaneous light measurements on top of the enclosure and above the surrounding structures. Even point measurements conducted on overcast days (Parent and Messier, 1996; Messier and Parent, 1997) can provide a realistic estimate of light reduction on the top of the enclosure relative to a completely open location (e.g., Pons et al., 1993), but longer-term continuous measurements at least over several days are recommended to improve the accuracy. Alternatively, hemispheric photographs can be taken above the enclosure and reduction of both direct and diffuse components of light assessed (Anderson, 1964; Pearcy, 1989; Grimmond et al., 2001). This initially for woodlands designed method can be improved for the use in urbanized areas by using simultaneously visible and near-infrared hemispheric photography (Osmond, 2009, 2010) or hemispheric CCD radiometers (Kuusk et al., 2002; Kuusk and Paas, 2007) that allow for better accounting of scattered light fluxes from vegetation and artificial structures.

### Enclosures with artificial light: Roles of illuminaire depreciation and distance from light source

In the case of wholly artificial light with a constant intensity of *Q*, the daily light integral incident to a given leaf (mol m^−2^ day^−1^) is the product of *Q*, and photoperiod length (Δ_P_, *h*):

(5) $$Q_{\text{int}}=Q\Delta_{\text{ P}}\text{3600}\cdot \text{1}{\text{0}}^{-\text{6}}$$

Once assessed, *Q*_int_ is typically assumed to be invariable. However, this does not consider that light intensity of any illumination source decreases in time due to a variety of reasons. The light output of lamps slowly decreases in time (lamp lumen depreciation), e.g., due to bulb wall darkening, phosphor exhaustion, filament depreciation, etc., that follow lamp-specific time kinetics. According to manufacturer’s (Philips, Osram, Sylvania, General Electric) specifications, the reduction of light level of most widely used gas discharge lamps during a typical experiments of 2–5 months is only 1–3%. However, the light output of fluorescent lamps decreases somewhat faster, 5–10% over 2–5 months (DiLaura et al., 2011). On the other hand, luminaire dirt depreciation can dramatically reduce light output if the luminaire surfaces are not regularly cleaned (e.g., Clark, 1966), but such effects cannot be considered *a posteriori* on the basis of information typically reported in plant acclimation studies. At most, we estimate that such problems can result in changes in light availability by 10–20%. On the other hand, changes in lamp spectra during lamp aging can importantly alter plant extension growth (Diffey, 1988), but again, this cannot be considered in retrospective analyses with any degree of accuracy.

**Figure 1:**
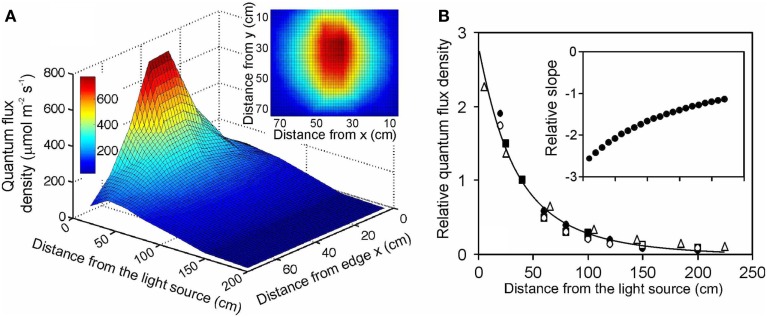
**Illustration of (A) the light field of a quartz metal halide lamp with opalized elliptical bulb (Philips Master HPI Plus Daylight 400 W) embedded in a rectangular low bay (length × width × height: 480 × 300 × 160 mm) Start SM HPI-TE40 luminaire (C Luce Srl., Truccazzano, Milan, Italy) and (B) the normalized change of light intensity for four different light sources: filled circles – the same light source as in (A) open squares – the same lamp, but embedded in a circular high bay (height of 548 mm and diameter of the luminaire window of 488 mm) Easy C Base HPI-BU luminaire (C Luce Srl.); open circles – quartz metal halide lamp with clear tubular bulb (Philips Master HPI-T Plus 400 W) in the Start SM luminaire; open triangles – fluorescent tube (Polylux XL, F36W/840, General Electric, Inc.) embedded in a Philips TMS022 luminaire (1225 × 80 × 56 mm)**. The light field was measured with a LI-190 quantum sensor (Li-Cor, Inc., Lincoln, Nebraska, USA) every 0.2 m from the lamp distance using a black squared board (0.8 m side length) with 64 regularly spaced measurement locations (altogether 7 heights, giving 448 measurements). The inset in **(A)** demonstrates a representative light field taken at 20 cm from the lamp surface (*x* and *y* correspond to the distance from the edge of the board). The relationship between lamp distance and light intensity in **(B)** was developed for averages measured for the central 30 cm × 30 cm area, and the data were normalized with respect to the measurements at 40 cm from the luminaire surface. For comparison, data for a high pressure sodium lamp with a clear tubular bulb (Philips, Master SON-T PIA Plus 600 W) from Buck-Sorlin et al. (2010) are also demonstrated (filled squares). The data in **(B)** were fitted Eq. 12, and the inset demonstrates changes in relative slope (m^−1^) calculated as $△y/(\bar{y}△x)$ where Δ*y* and Δ*x* are finite changes in the function value and argument, and $\bar{y}$ is the average function value over the given finite range.

A much larger source of uncertainty can result from the circumstance that the illumination source is at a finite distance *r* from the chamber floor. This is important as the intensity of light at any height, *h*, in the growth chamber without plants, *Q*_L_(*h*), is expected to decrease with the distance from the radiation source, *d* = *r − h*. For a point light source with completely isotropic radiation, the reduction of light intensity without any obstructing elements follows the inverse square law (Gates, 1980):

(6) $$Q_{\text{L}}\left(h\right)\propto \frac{Q_{0,\text{L}}}{{\left(r-h\right)}^{2}}$$

where *Q*_0,L_ is the light intensity at the lamp surface. In practice, light sources used for plant growth have finite sizes and are equipped with reflectors, and cannot therefore be considered isotropic point sources. For these light sources, the reduction of light intensity depends on the geometry of light source, e.g., bulb shape and reflector geometry. The reduction of light intensity in plant growth chambers also depends on the radiative characteristics of growth chamber walls, and in practice, empirical functions need to be derived to describe the actual distance-dependent reductions in light intensity (see Measurement of Light Fields of Common Lamps Used in Plant Growth Studies, Figure 1).

When the plants are enclosed in the chamber, the light available to the leaves at different depths in the canopy is driven both by the distance from the light source and cumulative leaf area from canopy top to given canopy depth, *L*_c_(*h*). In a most simple form following the Lambert–Beer law (Monsi and Saeki, 1953), the light intensity incident to the foliage at given height *h*, *Q*(*h*), is:

(7) $$Q\left(h\right)=Q_{\text{L}}\left(h\right)e^{-k_{1}L_{\text{c}}\left(h\right)}$$

where *k*_1_ is the extinction coefficient for the artificial light, and *Q*_L_(*h*) (Eq. 6) provides a light intensity incident to a given layer in the canopy. The key implication of this equation is that the light gradient is much stronger with artificial light than with natural illumination, for which *r *− *h* is very large relative to *h*, and thus, *Q*_L_ is a constant equal to light intensity above the canopy (standard Lambert–Beer model). Another important implication for calculating the average light integral at different depths in the canopy is that the light integral will inevitably change for growing plants continuously increasing in height unless the distance between the light source(s) and plants is not kept constant. On the other hand, raising the height of the light source(s) to maintain the light integral for the top of the canopy, will unavoidably reduce the light for the bottom of the canopy. Thus, estimation of an average *Q*_int_ gradient through the canopy would require frequent measurements of light profiles within the developing canopy. Additional complications arise from reflectance of chamber walls, non-homogeneous incident light fields, etc., and in practice, highly complex light gradients can occur within growth chambers such that Eq. 6 is a crude simplification (Chelle et al., 2007; Delepoulle et al., 2009) and prediction of the dynamics of the light field in growing plant stands can be an highly tedious task (Chelle et al., 2004, 2007; Delepoulle et al., 2008). In fact, each chamber can even be considered a unique radiative transfer system, and numerical approaches may be needed for precise description of light environment (Chelle, 2005; Chelle et al., 2007). Parameterization of such numerical models requires highly detailed information on chamber, plant, and lighting geometry that is typically not available. In fact, even the distances between the light source and chamber floor and vegetation top were not reported in any of the studies investigating light gradients in plants grown with artificial illumination (Table 1 for the studies). In the current study, a simplified method is developed based first on estimation of a generalized light intensity vs. distance relationship and using this relationship to determine relevant light field characteristics for the specific studies (see Measurement of Light Fields of Common Lamps Used in Plant Growth Studies,” and “Estimation of Dynamic Changes in Light Profiles in Growth Chambers).

**Table 1:** **Analyzed studies investigating within-canopy plasticity in foliage dry mass per unit area (*M*_A_), nitrogen content per area (*N*_A_), and photosynthetic capacity (*A*_max_) under different treatments in artificial environments**.

Reference	Species	Life form	Treatment type	Number of treatments	Data availability		
					*M*_A_	*N*_A_	*A*_max_
**EXPERIMENTS WITH NATURAL LIGHTING (GREENHOUSES, MICROCOSMS)**							
Ackerly and Bazzaz (1995)	*Heliocarpus appendiculatus*	Evergreen tree	N nutrition/shading	4			Y
Acock et al. (1978)	*Lycopersicon esculentum*	Annual herb	No treatments	1			Y
Anten and Ackerly (2001)	*Chamaedorea elegans*	Evergreen shrub	Defoliation	3			Y
Boonman (2006), Boonman et al. (2006), Boonman et al. (2007), Boonman et al. (2009)	*Nicotiana tabacum*	Perennial herb	Canopy density	3	Y	Y	Y
Evans (1993a,b)	*Medicago sativa*	Perennial herb	Replicates in time	3	Y	Y	Y
Forstreuter (1995, 1996)	*Fagus sylvatica*	Deciduous tree	CO_2_ concentration	2	Y	Y	Y
Hirose et al. (1988)	*Lysimachia vulgaris*	Perennial herb	Canopy density	2	Y	Y	
Pons and Jordi (1998), Pons and Anten (2004)	*Lysimachia vulgaris*	Perennial herb	N nutrition/canopy density	4		Y	Y
Schieving et al. (1992)	*Carex acutiformis*	Perennial grass	Canopy density	2		Y	Y
Sims et al. (1999)	*Helianthus annuus*	Annual herb	CO_2_ concentration	2		Y	Y
**EXPERIMENTS WITH NATURAL AND SUPPLEMENTAL LIGHTING (GREENHOUSES)**							
Dreccer et al. (2000)	*Triticum aestivum*	Annual grass	N nutrition/canopy density/replicates in time	16		Y	Y
Pettersen et al. (2010)	*Cucumis sativus*	Annual vine	Intracanopy lighting	3			Y
Pons et al. (1993)	*Carex acutiformis*	Perennial grass	N nutrition/intracanopy lighting	4		Y	Y
Trouwborst et al. (2010)	*Cucumis sativus*	Annual vine	Intracanopy lighting	2	Y	Y	Y
Xu et al. (1997)	*Lycopersicon esculentum*	Annual herb	No treatment	1			Y
**EXPERIMENTS WITH ARTIFICIAL LIGHTING (GROWTH CHAMBERS)**							
Gutschick and Wiegel (1988), Pushnik et al. (1988), Gutschick and Cunningham (1989)	*Medicago sativa*	Perennial herb	No treatment	1	Y		
Lötscher et al. (2003)	*Medicago sativa*	Perennial herb	Stand density/interspecific competition	5		Y	Y
Lötscher et al. (2003)	*Dactylis glomerata*	Perennial grass	Stand density/interspecific competition	4		Y	
Lötscher et al. (2003)	*Taraxacum officinale*	Perennial herb	Stand density/interspecific competition	5		Y	
Pons et al. (1993)	*Brachypodium pinnatum*	Perennial grass	Canopy density/intracanopy lighting	3		Y	Y

### Enclosures with combined natural and supplemental artificial light

The situation is even more complex when plants are simultaneously exposed to both natural and artificial light as is common practice in greenhouses operated over the entire year in seasonal climates. The total light input to the plant under such circumstances is the sum of artificial and natural light. As discussed above, the intensity of artificial light sources varies relatively little, and the major source of variation of light incident to plant foliage is due to the distance between plants and the light source. In contrast, natural light input strongly varies between days and seasons and its intensity is also importantly affected by enclosure transmittance. Thus, it is difficult to obtain a constant daily light integral incident to vegetation over a certain time period (Ferentinos et al., 2000; Ferentinos and Albright, 2005; Seginer et al., 2006; Ioslovich, 2009). In studies on within-canopy light acclimation using combined lighting, no attempt has been made to compensate for temporal variations in natural light by changing the artificial light input. Thus, an effort is needed to obtain the integrated light over the standardized time period.

For combined lighting, the incident quantum flux density by artificial light source is given by Eq. 7, and an analogous equation based on Lambert–Beer law can be employed to describe the reduction of natural light within the canopy. For both components of light:

(8) $$Q\left(h\right)=Q_{\text{L}}\left(h\right){\text{e}}^{-k_{1}L_{\text{c}}}+\kappa_{\text{Q}}Q_{\text{ab}}{\text{e}}^{-k_{2}L_{\text{c}}}$$

The effective light extinction coefficients for artificial (*k*_1_) and natural (*k*_2_) light can be different due to different lighting geometries, and *k*_2_ varies during the day and season due to differences in solar position and sky conditions. As we are interested in integrated light incident to the plant foliage, *Q*_int_(*h*), we rewrite the equation as:

(9) $$Q_{\mathrm{int}}\left(h\right)=Q_{\text{L}}\left(h\right){\text{e}}^{-k_{1}L_{\text{c}}}\Delta_{\text{P}}3600\cdot 10^{{}_{-6}}+\kappa_{\text{Q},\text{av}}Q_{\text{int},\text{ab}}{\text{e}}^{-k_{\text{int}}L_{\text{c}}}$$

where *Q*_int,ab_ is the average integrated quantum flux density above the enclosure, and *k*_int_ is the effective canopy extinction coefficient characterizing the penetration of integrated radiation during the period of interest. The total average integrated quantum flux density at the top of the canopy (*L*_c_ = 0) at height *h*_0_ is given as:

(10) $$Q_{\mathrm{int}}\left(h_{0}\right)=Q_{\text{L}}\left(h_{0}\right)\Delta_{\text{P}}3600\cdot 10^{{}_{-6}}+\kappa_{\text{Q},\text{av}}Q_{\text{ab},\text{int}},$$

Equations 9 and 10 predict that the contribution of artificial light decreases with increasing depth in the canopy due to reduction of artificial light intensity with the distance from the light source. This needs to be considered when estimating dynamic changes in light during plant growth. The relative quantum flux density, *R*_Q_, provided by most studies is *Q*_int_(*h*)/*Q*_int_(*h*_0_), and is driven by the share of light at the top of canopy between artificial and relative light, by the distance from canopy top and light extinction by vegetation (Eqs 9 and 10).

## Materials and Methods

### Studies on within-canopy variations in key foliage structural, chemical, and physiological traits in artificial environments

A thorough literature survey was carried out to find studies on acclimation of key foliage traits to within-canopy light gradients in artificial environments. In particular, focusing on studies investigating within-canopy variations in leaf dry mass per unit area (*M*_A_), leaf nitrogen content per area (*N*_A_), and photosynthetic capacity (*A*_max_). Altogether data from 25 papers were included in the analysis providing information for 70 study × species × treatment combinations (Table 1).

### Estimation of enclosure light transmittances

When average transmittance values of the enclosure, κ_Q,av_, were available in the original study, we used these values: (Anten and Ackerly, 2001) – 0.27; (Pons et al., 1993) – 0.48; (Dreccer et al., 2000) – 0.60; (Trouwborst et al., 2010) – 0.62. However, unrealistic values of κ_Q,av_ were reported in some studies (e.g., a value of 0.85 in Sims et al., 1999), suggesting that measurements of transmittance corresponding to mid-day only were used, thereby ignoring the angular dependence of transmittance [see Theory: Light in Manipulated Environments (Microcosms, Greenhouses, Growth Chambers)]. In these cases, and when κ_Q,av_ was not available, it was derived according to Eq. 4. In derivation of κ_Q,av_, data on transmittance for direct light at different angles of incidence, κ_Q,dir_(Φ), and for diffuse light, κ_Q,dif_, for various enclosure wall materials were taken from Nijskens et al. (1985), Altuglas International (2000), Papadakis et al. (2000) and Pollet and Pieters (2002). For average κ_Q,dir,av_ over the period used to estimate *Q*_int_ (see Enclosures with Natural Lighting), the daily time-course of quantum flux density at specific times of the year and geographic latitude was predicted according to Campbell and Norman (1998), and an average κ_Q,dir,av_ corresponding to the time of the study was derived. If the values of the fraction of diffuse light over the study period, *f*_dif_, had been reported in the original studies, these could have been used in Eq. 4. However, as this information was not available in any of the studies, a global annual average value of 0.42 (Mercado et al., 2009) was used, and κ_Q,av_ calculated.

### Measurement of light fields of common lamps used in plant growth studies

To evaluate the overall effect of changes of the distance between light source and vegetation, and develop a generalized distance vs. light intensity relationship, light fields were measured for four characteristic light sources used in plant growth studies: (1) a quartz metal halide lamp with elliptical opalized bulb (Philips Master HPI Plus Daylight 400 W) embedded in a rectangular low bay (length × width × height: 480 × 300 × 160 mm) Start SM HPI-TE40 luminaire (C Luce Srl., Truccazzano, Milan, Italy); (2) the same lamp, but embedded in a circular high bay (height 548 mm and the diameter of the luminaire window 488 mm) Easy C Base HPI-BU luminaire (C Luce Srl.); (3) quartz metal halide lamp with clear tubular bulb (Philips Master HPI-T Plus 400 W) in the Start SM luminaire; (4) fluorescent tube (Polylux XL, F36W/840, General Electric, Inc.) embedded in Philips TMS022 luminaire (1225 × 80 × 56 mm). The light field was measured with a LI-190 quantum sensor (Li-Cor, Inc., Lincoln, Nebraska, USA) every 0.2 m from the luminaire surface using a black squared board (0.8 m side length) with 64 regularly spaced measurement locations (Figure 1A). For each lamp, best fit relationships between distance from the luminaire and light intensity were developed for average quantum flux densities measured for the central 30 cm × 30 cm area. Using different areas from 10 cm × 10 cm to 60 cm × 60 cm for averaging did not qualitatively change the shape of the relationship. Although multiple light sources may be simultaneously used with overlapping light fields at the bottom of the enclosure, the horizontal distance of the lamps is typically adjusted such that the light fields overlap relatively far from the light source (Chelle et al., 2007; Delepoulle et al., 2009; Buck-Sorlin et al., 2010). In our study, we estimated that for multiple low bay illuminaires spaced 0.5 m apart, light intensity for the central 30 cm × 30 cm part used for averaging in our study (Figure 1B) is 1–2% (0.2 m from the lamp) to 20–30% (0.8 m from the lamp) larger than for a single luminaire (data not shown). Thus, this simulation suggests that strong light gradients are also present with multiple light sources. Clearly light gradients and the uniformity of light field can be importantly altered by varying the number of lamps and their spacing, and for smaller enclosures by altering the wall reflectance characteristics (Chelle et al., 2007; Delepoulle et al., 2008, 2009; Buck-Sorlin et al., 2010), but these effects cannot be accurately considered in retrospective analyses.

Starting from the classical inverse square relationship (Eq. 6), various inverse power models were tested to describe the reduction of light intensity with distance *d* = *r − h* from the light source, *Q*(*d*). An empirical model in the form

(11) $$Q\left(d\right)=\frac{g_{1}}{g_{2}{\left(d-g_{3}\right)}^{g_{4}}},$$

where *g*_1_–*g*_4_ are empirical parameters, was found to best describe the reduction of light intensity with the distance from the light source for all different light sources (*r*^2^ = 0.995–0.9999, Table 2).

**Table 2:** **Distance-dependent changes in light intensity for four different plant growth light sources**.

Light source	Regression coefficients (Eq. 11)				*r*^2^	Light intensity at *d*_s_ (0.4 m)*(μmol m^−2^ s^−1^)
	*a*_1_	*a*_2_	*a*_3_	*a*_4_	
Philips Master HPI Plus Daylight 400 W (opalized elliptical bulb) in a rectangular low bay illuminaire	924	1.40	0.739	3.30	0.9994	430
The same lamp in a circular high bay luminaire	628	11.9	0	1.67	0.9998	242
Philips Master HPI-T Plus 400 W (clear tubular bulb)	1095	0.98	1.03	4.23	0.995	246
Polylux XL, F36W/840 fluorescent tube in Philips TMS022 luminaire	53.1	0.438	0.89	2.58	0.9999	63

*d_s_ refers to standard height used to normalize the distance vs. light intensity relationships (Eq. 11).

The light intensity of different lamps differs due to lamp output and illuminaire characteristics, determining the effective solid angle of light, and resulting in different parameter values *g*_1_–*g*_4_ of Eq. 11 (Table 2). Therefore, the data were normalized with respect to the measurements conducted at 0.4 m from the lamp surface (standard distance, *d*_s_), and a best fit relationship was developed for all standardized data pooled (*r*^2^ = 0.975, *P* < 0.001, Figure 1B):

(12) $$Q\left(d\right)/Q\left(d_{\text{s}}\right)=\frac{21.3}{1.683{\left(d-1.40\right)}^{4.31}},$$

where *d* is in m. All lamps closely fit this relationship, demonstrating that it adequately captured the shape of the distance-dependent reductions of light. This equation also fit an independent dataset for a high pressure sodium lamp with a clear tubular bulb (Philips, Master SON-T PIA Plus 600 W) from Buck-Sorlin et al. (2010; Figure 1B).

Overall, it is hard to assess the error in derivation of *a posteriori* estimates of lamp height vs. intensity relationships. We suggest that the degree of deviation of data points for different lamps from the best fit general relationship (Figure 1B) can be considered as a measure of accuracy of this method. The deviation for different lamps at heights different from the standard height was 3–50%, on average 22% for all data points (Figure 1B).

### Estimation of dynamic changes in light profiles in growth chambers

The generalized relationship between the distance from the lamp and quantum flux density relative to the measurement at standardized height *d*_s_ of 0.4 m (Eq. 12) was further employed to determine the actual distance-dependent light gradient in specific studies. The studies reported the quantum flux density at the top of the vegetation, *Q*(*h*_0_), but did not report the distance between the lamp and vegetation (*r *− *h*_0_). The key issue in using Eq. 12 for prediction of the actual light gradient is to determine this distance that allows for conversion of the relative dependence to absolute scale going through *Q*(*h*_0_). This was achieved by considering that the light intensity at the standard height *d*_s_ of a given lamp, *Q*_1_(*d*_s_), together with the information of the overall light output of the lamp (μmol s^−1^) can be used to estimate the light intensity at a given height for another lamp with different output. The photosynthetic quantum output of a lamp is given by its lumen output, Φ, and its lumen/quantum conversion factor, χ (lm s μmol^−1^). Thus, for another lamp, the light intensity at the standard height, *Q*_2_(*d*_s_), is given as:

(13) $$Q_{2}\left(d_{\text{s}}\right)=\frac{\Phi_{2}Q_{1}\left(d_{\text{s}}\right)\chi_{1}}{\chi_{2}\Phi_{1}},$$

where Φ_1_ is the lumen output of the first and Φ_2_ of the second lamp, and χ_1_ and χ_2_ are the photometric to quantum unit conversion factors for the first and the second lamp. We used Philips SON-T-600 W as an independent standard lamp (the light intensity at a distance of 0.4 m based on the data of Buck-Sorlin et al., 2010). Using this estimate, we calculated the light intensity at the standardized height for all the light sources used in different studies. The lumen outputs were taken from manufacturers specifications and χ values from Thimijan and Heins (1983). The estimates of *Q*(*d*_s_) along with the information on *Q*(*h*_0_) and vegetation height (*h*_0_) reported in original studies were further used to compute *r *− *h*_0_ and *r* from Eq. 12. To quantitatively evaluate the importance of considering the distance from the lamp, we estimated the change in light intensity at the top of plants for stands of hypothetical height (Gutschick and Wiegel, 1988; Gutschick and Cunningham, 1989; Pons et al., 1993; Lötscher et al., 2003; Pettersen et al., 2010).

To gain further insight into the effects of distance-dependent changes in light intensity, we calculated the integrated quantum flux density during foliage growth (*Q*_int,G_) using the study-specific relationships between light intensity and distance. For canopies developing mainly from the top such as herbs and graminoids primarily carrying leaves on parent shoots and tillers with few elongated basal leaves, the *Q*_int,G_ was simply taken as the integrated quantum flux density at the vegetation height at a particular time. We acknowledge that this is a simplified estimate as it does not consider elongation of older phytomers and growth of new tillers into the expanding canopy. Nevertheless, phyllochron (phytomer formation) is a relatively non-plastic trait, and new leaves are generally not emerging from the sheath of the previous leaf until the older leaf has attained ca. 50% of its final length (Schnyder et al., 1987; Nelson, 2000). Given also the erect habit of grass foliage, we argue that *Q*_int,G_ estimated this way for tillering grasses provides a realistic estimate of light environment during the formation of the bulk of the leaves. Average quantum flux density during foliage lifetime (*Q*_int,av_) was further calculated as the average of *Q*_int,G_ and integrated quantum flux density at the time of the measurements (*Q*_int,C_). Calculating the average value this way assumes that the amount of shading due to canopy development increases linearly in time.

In contrast, in graminoids with primarily basal growth such as tuft-forming *Carex* species, determination of *Q*_int,G_ is more complex. In these species, leaves continuously extend from basal leaf portions. The leaf parts formed earlier have been exposed to higher irradiance than the leaf parts developed later when there is already considerable shading by upper canopy elements. In growth chambers, the maximum irradiance experienced by uppermost leaves during their growth is that provided by the light source to the bottom of the chamber, *Q*_int,B_. For the next lower section, as a first approximation, *Q*_int,G_ is the product of *Q*_int,B_ and current relative light at that layer, *R*_Q_. However, as the upper layers have not yet been fully developed at the time when the given lower layer had developed, we estimated *Q*_int,G_ as the product of *Q*_int,B_ and the average of *R*_Q_ of the next two upper layers (typically 10 cm thick layers as sampled in original studies). With leaf elongation, leaf *Q*_int_ continuously increases as foliage becomes positioned closer to the light source. Given a layered canopy, and assuming a constant leaf elongation rate, average *Q*_int_ during leaf lifetime for given leaf layer *i* was estimated as:

(14) $$Q_{\mathrm{int},\text{av}}\text{(}i\text{)=}\frac{\sum_{n=x}^{n=i}{\text{Q}}_{\text{int},\text{L}}(n)}{x+1}\frac{\sum_{n=i}^{n=1}R_{Q}(n)}{l},$$

where *Q*_int,L_(*n*) is the integrated quantum flux density without plants for layer *n*, *x* is the number of leaf layers below the current layer *i*, and *l* is the sum of given and upper leaf layers used for calculating average *R*_Q_ that characterizes the shading of developing canopy (*l* = 3 for all, except for the uppermost layer: *l* = 1, and for the layer below the uppermost layer: *l* = 2).

### Estimation of changes in light profiles for studies with combined natural and supplemental lighting

Only one study (Pons et al., 1993) reported the variation in the contributions of artificial and natural light along the canopy. Other studies provided data on relative light or on integrated light based on the sum of supplemental and natural light. For these studies, we first estimated the height profile of artificial light from *Q*(*h*_0_) and canopy height as for studies with artificial light only. Thereafter, we evaluated the overall share of incident light between natural and artificial light. For natural light, if available, we used the estimates provided by original studies (Pons et al., 1993; Trouwborst et al., 2010). For other studies we derived an estimate as described in the Section “Estimation of Integrated Incident Light Over A Standardized Time Period” and corrected this for greenhouse transmittance. For studies reporting leaf area index profiles, we fit Eq. 9 to the data by minimizing the sum of squares between estimated and measured *Q*_int_ or *R*_Q_. As fitting both the extinction coefficients *k*_1_ and *k*_int_ (Eq. 9) simultaneously yielded often unrealistic estimates for the extinction coefficient(s), a single extinction coefficient for both artificial and natural light was used. If leaf area index profiles were lacking, we used canopy light profiles in relation to depth into the canopy, *h*_0_ − *h*, instead of *L*_c_, and fitted an apparent extinction coefficient, *w*_j_ $\left(Q_{\mathrm{int}}(h)\text{α }{\text{e}}^{-w_{j}(h_{0}-h)}\right)$.

Two studies included treatments with intracanopy lighting (Pettersen et al., 2010; Trouwborst et al., 2010). For these treatments, side illumination was included in the height profile of artificial light *Q*_L_(*h*), and Eq. 9 was re-fitted as above for all other treatments.

The integrated light during foliage development, *Q*_int,G_, is the sum of artificial and natural light at the time of foliage development. The contribution of artificial light for all species was found as for the studies with artificial light only. Natural light during foliage formation for herbs and tillering grasses growing from apex is equal to that at the top of vegetation during the study. However, for turf-forming graminoids, the intensity of natural light during growth at leaf base decreases due to canopy development. For these species, natural light was taken as the average of light in the current and the next upper layer at the time of sampling as with artificial light.

For species with apical growth, average light during leaf lifetime, *Q*_int,av_, was found as the average of *Q*_int,G_ and *Q*_int,C_. For species with basal growth, *Q*_int,G_ for artificial light was computed by Eq. 14, while *Q*_int,G_ for natural light was taken as the average of light in the current and the next two upper layers at the time of sampling.

### Estimation of integrated incident light over a standardized time period

For artificial light, the leaf light availability estimates, *Q*_int,C_, *Q*_int,G_, and *Q*_int,av_, are derived assuming that lamp light output does not change in time (Eq. 5). However, for studies with natural and with supplemental lighting, inherent variation in natural light intensity incident to enclosures due to day-to-day and seasonal variations in a given study and among studies due to different integration periods and times of measurement implies that it is important to derive a standardized estimate of incident integrated natural light. Typically, in acclimation studies, average integrated quantum flux density of 3–90 days prior to foliage sampling is used as an estimate of leaf light availability (Klein et al., 1991; Barker, 1996; Le Roux et al., 1999; Koike et al., 2001; Casella and Ceulemans, 2002; Fleck et al., 2003; Niinemets et al., 2004). On the other hand, it has been demonstrated that stable values of *Q*_int_ vs. leaf trait relationships are observed with average integrated light of 30–60 days following leaf formation (Niinemets et al., 2004). We estimated the dates of budburst and leaf formation based on information on the dates of the start of the experiment, dates of seed germination, and whenever pertinent, on the rate of leaf formation and longevity, reported in original studies. We further estimated a standardized average *Q*_int_ for 50 days following budburst or seed germination for all studies. This estimate was derived on the basis of a global gridded database of shortwave daily radiation data from the NASA Langley Research Center Global Energy and Water Cycle Experiment (GEWEX 3.0, http://www.gewex.org/srbdata.htm; Cox et al., 2006; Fritsen et al., 2011; Stackhouse et al., 2011). Dates of the experiments and coordinates of study sites were extracted from the papers and shortwave radiation at each pixel was estimated from the 1°× 1° gridded data. Shortwave radiation data were further converted to PAR (photosynthetically active radiation, W m^−2^) using a conversion factor of 0.434 (Ross and Sulev, 2000). Thereafter, PAR data were converted to photosynthetic quantum flux density using a representative *Q*/PAR conversion factor of 4.56 μmol J^−1^ (Dye, 2004). Finally, average integrated values of *Q*_int_ for 50 days after leaf formation were derived.

### Analyses of the effects of different light estimates on foliage structural, chemical, and physiological traits

We used linear and non-linear regressions in the form *y* = *a*_1_*x*^b1*c*^ and *y* = *a*_2_Ln(*x*) + *b*_2_ to fit the statistical relationships among leaf traits and current, growth and average integrated quantum flux density for all studies. All three functions have often been employed in investigating the effects of light on foliage traits (e.g., Hirose et al., 1988; Niinemets et al., 2004, 2010; Pons and Anten, 2004).

The studies analyzed often included multiple treatments (Table 1), but the correlations with foliage traits within any specific treatment were generally similar for *Q*_int,C_, *Q*_int,G_, and *Q*_int,av_ (data not shown). However, treatments importantly altered canopy height and foliage distribution, and thus, leaf “light history” at given *Q*_int,C_. To assess the fundamental differences between different light estimates, we pooled treatments within a given study to assess the explanatory power of different light estimates in determining foliage traits.

As a measure of the goodness of fit, the explained variance, *r*^2^, was used. Average *r*^2^ values across the studies for given trait with *Q*_int,C_, *Q*_int,G_, or *Q*_int,av_, and for thee different types of regressions were compared by paired samples *t*-tests. Only two studies with artificial and combined lighting (Gutschick and Wiegel, 1988; Trouwborst et al., 2010) provided information on *M*_A_, and thus, only the effect of different light estimates on *N*_A_ and *A*_max_ was tested.

### Estimation of the bias introduced in light vs. foliage trait relationships by use of apparent relative light

To estimate the potential bias of using relative light inside the enclosure, *R*_Q,A_, instead of light relative to full sun, *R*_Q_, typically estimated in field experiments (*R*_Q_ = κ_Q,av_*R*_Q,A_), in the regressions with plant traits, we fitted linear (*y* = *ax* + *b*, where *x* is either *R*_Q_ or *R*_Q,A_), and non-linear power (*y* = *a*_1_*x*^c^) and logarithmic (*y* = *a*_2_Ln(*x*) + *b*_2_) regressions to the data and derived regression parameters for key leaf traits, *M*_A_, *N*_A_, and *A*_max_ in each study. As here we were interested in potential bias in regression parameters within and across studies, different treatments were fitted separately. For all regression parameters, we calculated the relative change in a given parameter value (*p*_j_) due to use of *R*_Q,A_ instead of *R*_Q_ as:

(15) $$\upsilon =\frac{p_{\text{j}}\left(R_{\text{Q}}\right)-p_{\text{j}}\left(R_{\text{Q},\text{A}}\right)}{p_{\text{j}}\left(R_{\text{Q}}\right)}.$$

For linear regressions, *y* = *a*_,RQ_*R*_Q_ + *b* = *a*_,RQA_*R*_Q,A_ + *b *=*a*_,RQA_*R*_Q_/κ_Q,av_ + *b*, where *a*_,RQ_ is the slope for fits with *R*_Q_, and *a*_,RQA_ is the slope for fits with *R*_Q,A_. From this, *b* is the same for fits with both *R*_Q_ and *R*_Q,A_, and the relative change in the slope *a*, υ, is equal to 1 − κ_Q,av_.

For power regression, $y=a_{1,\text{RQ}}R_{Q}{\;}^{c}\;=\;a_{1,\text{RQA}}R_{\text{Q,A}}{\;}^{c}\;=\;a_{1,\text{RQA}}{\left(R_{\text{Q}}/{\text{κ}}_{\text{Q,av}}\right)}^{c}$ where *a*_1,RQ_ is the regression slope for fits with *R*_Q_, and *a*_1,RQA_ for fits with *R*_Q,A_. Thus, the relative change for the slope is:

(16) $$\upsilon_{a1}=1-{\text{κ}}_{\text{Q},\text{av}}{\;}^{c}.$$

Finally, for logarithmic regression, *y* = *a*_2_Ln(*R*_Q_) + *b*_2,RQ_ = *a*_2_Ln(*R*_Q,A_) + *b*_2,RQA_ = *a*_2_Ln(*R*_Q_/κ_Q,av_) + *b*_2,RQA_ where *b*_2,RQ_ is the intercept for fits with *R*_Q_ and *b*_2,RQA_ the slope for fits with *R*_Q,A_. Accordingly, the intercept *b*_2,RQA_ is equal to *b*_2,RQ_ + *a*_2_Ln(κ_Q,av_). From this, the relative change for the intercept is given as:

(17) $$\upsilon_{\text{b}2}=\frac{-a_{2}\text{Ln}\left(\kappa_{\text{Q},\text{av}}\right)}{b_{2,\text{RQ}}}.$$

## Results

### Natural light transmittance in greenhouses

For enclosures with natural illumination only, average transmittance (κ_Q,av_, Eq. 4) values obtained were between 0.46 and 0.84, on average (±SE) 0.648 ± 0.042 (Table 3). Only four studies (Pons and Jordi, 1998; Sims et al., 1999; Anten and Ackerly, 2001; Pons and Anten, 2004) reported a value for enclosure transmittance, and these values varied between 0.27 and 0.85 (average ± SE = 0.57 ± 0.16). The exceptional value of 0.27 in (Anten and Ackerly, 2001) refers to an enclosure painted white to result in lower light intensities than in typical greenhouses (Anten and Ackerly, 2001), while the high value of 0.85 for two-layer acrylic greenhouse roof and polyethylene tops of EcoCELL mesocosms (Griffin et al., 1996) employed in Sims et al. (1999) likely reflected mid-day estimations with low solar incidence angle.

**Table 3:** Enclosure transmittances and potential biases (Eq. 15) introduced in the non-linear regressions of leaf dry mass per unit area (*M*_A_), nitrogen content per area (*N*_A_), and photosynthetic capacity per area (*A*_max_) with apparent relative light (*R*_Q,A_)* in studies with natural illumination in greenhouses and microcosms.

Study	Species	Treatment	Enclosure transmittance	Relative underestimation of the slope of $y=a_{2}R_{Q,A}{\;}^{c}$		
				*y* = *M*_A_	*y* = *N*_A_	*y* = *A*_max_
Ackerly and Bazzaz (1995)	*Heliocarpus appendiculatus*	High light, low N	0.45			0.46
	*Heliocarpus appendiculatus*	Low light, low N	0.45			0.58
	*Heliocarpus appendiculatus*	High light, high N	0.45			0.42
	*Heliocarpus appendiculatus*	Low light, high N	0.45			0.43
Acock et al. (1978)	*Lycopersicon esculentum*	No treatment	0.82			0.10
Anten and Ackerly (2001)	*Chamaedorea elegans*	All data pooled	0.27			0.91
Boonman (2006), Boonman et al. (2006), Boonman et al. (2007), Boonman et al. (2009)	*Nicotiana tabacum*	Low density	0.62		0.18	
	*Nicotiana tabacum*	Medium density	0.62		0.057	0.40
	*Nicotiana tabacum*	High density	0.62	0.11	0.11	0.26
Evans (1993a,b)	*Medicago sativa*	Replicate in time	0.84s	0.028	0.025	0.084
	*Medicago sativa*	Replicate in time	0.83	0.051	0.036	0.055
	*Medicago sativa*	Replicate in time	0.84	0.041	0.030	0.057
Forstreuter (1995, 1996)	*Fagus sylvatica*	Ambient CO_2_	0.77	0.042	0.053	0.031
	*Fagus sylvatica*	Elevated CO_2_	0.77	0.051	0.061	0.063
Hirose et al. (1988)	*Lysimachia vulgaris*	Open stand	0.53	0.33	0.33	
	*Lysimachia vulgaris*	Dense stand	0.53	0.13	0.12	
Pons and Jordi (1998), Pons and Anten (2004)	*Lysimachia vulgaris*	Low N, high density	0.62		0.16	0.25
	*Lysimachia vulgaris*	Low N, low density	0.62		0.31	0.31
	*Lysimachia vulgaris*	High N, high density	0.62		0.11	0.17
	*Lysimachia vulgaris*	High N, low density	0.62		0.19	0.049
Schieving et al. (1992)	*Carex acutiformis*	High density	0.62		0.047	0.31
	*Carex acutiformis*	Low density	0.62		0.12	0.50
Sims et al. (1999)	*Helianthus annuus*	Ambient CO_2_	0.59		0.18	0.16
	*Helianthus annuus*	Elevated CO_2_	0.59		0.15	0.17

*Relative apparent light, *R*_Q,A_, refers to the ratio of integrated quantum flux density (*Q*_int_) incident to given leaves to *Q*_int_ above the plants. Due to limited light transmittance of enclosure (κ_Q,av_), *R*_Q,A_ is higher than the integrated light expressed relative to full sunlight outdoors *R*_Q_ = κ_Q,av_*R*_Q,A_.

For greenhouses with supplemental lighting (Pons et al., 1993; Dreccer et al., 2000; Pettersen et al., 2010; Trouwborst et al., 2010), the range of κ_Q,av_ was 0.48–0.62. For these studies, the only value derived was for Pettersen et al. (2010; κ_Q,av_ = 0.57). The values of κ_Q,av_ were employed to convert the *R*_Q,A_ values to corresponding estimates of *R*_Q_ (Eq. 3), and in analyzing the bias in light vs. leaf structure regressions (Eqs 15–17).

### Changes in light gradients in experiments with artificial lighting

After adjusting the standardized distance vs. light intensity curves to specific studies (Eq. 12, see Measurement of Light Fields of Common Lamps Used in Plant Growth Studies and Estimation of Dynamic Changes in Light Profiles in Growth Chambers), light intensity at the top of canopies with varying height, *h*_0_, could be determined. These relationships predicted that an hypothetical 50% shorter stand (0.125–0.95 m shorter) compared with the stand at the time of measurements (0.25–1.90 m; Gutschick and Wiegel, 1988; Gutschick and Cunningham, 1989; Pons et al., 1993; Lötscher et al., 2003; Pettersen et al., 2010) had 1.3- to 5.4-fold lower *Q*(*h*_0_). For a given reduction of stand height of 0.2 m, the reduction in light intensity was 1.4- to 3.1-fold, and a 0.5-m reduction in stand height (for stands taller than 0.5 m) was predicted to lead to 2.3- to 3.7-fold lower *Q*(*h*_0_). As the reduction of light intensity with distance from the luminaire, d*Q*/d*d*, becomes progressively less with increasing the distance (inset in Figure 1B), this variation in the reduction of light for a certain reduction of stand height reflects the study-to-study differences in the lamp to vegetation distance at the time of sampling.

**Figure 2:**
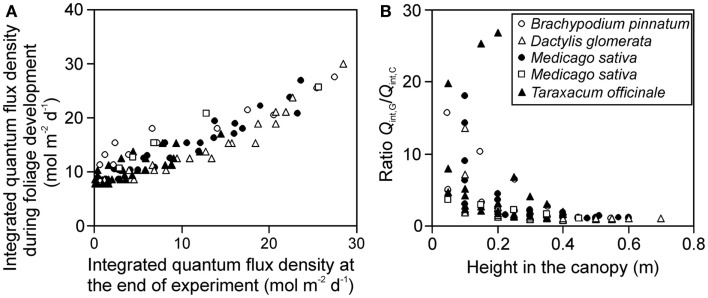
**Correlations between the integrated quantum flux density during growth (*Q*_int,G_) and at the time of measurements (current, *Q*_int,C_) (A), and the ratio of *Q*_int,G_/*Q*_int,C_ in relation to leaf height within the canopy (B) across studies conducted in growth chambers with artificial light only**. *Q*_int,G_ was determined as described in Materials and Methods (see Estimation of dynamic changes in light profiles in growth chambers and Estimation of Changes in Light Profiles for Studies with Combined Natural and Supplemental Lighting) after retrospective determination of plant height vs. incident quantum flux density relationships (see Measurement of Light Fields of Common Lamps Used in Plant Growth Studies and Estimation of dynamic changes in light profiles in growth chambers”). Data for *Brachypodium pinnatum* are from Pons et al. (1993), data for *Dactylis glomerata*, *Medicago sativa* (filled circles) and *Taraxacum officinale* are from Lötscher et al. (2003). Data for another experiment in *Medicago sativa* (open squares) are from Gutschick et al. (Gutschick and Wiegel, 1988; Pushnik et al., 1988; Gutschick and Cunningham, 1989).

The gradient in integrated light during foliage development (*Q*_int,G_) provides further insight into the effects of varying distance between the lamp and plant due to differences in leaf height. The ratio of *Q*_int,G_ at the leaves developing last at the top to *Q*_int,G_ at the bottom of canopy at the leaves developing first (for herbs and tillering grasses), varied between 1.8 and 6.4. The gradient in integrated light at the time of measurements (*Q*_int,C_) varied from 4.6 to 47 across the stands, being 1.4- to 12-fold larger than the gradient in *Q*_int,G_. Both *Q*_int,G_ and *Q*_int,C_ were correlated across the studies (Figure 2A), but the correlation was scattered and the ratio *Q*_int,G_/*Q*_int,C_ increased with decreasing height in the canopy (Figure 2B), indicating that for leaves developing first at the bottom of the canopy, the difference in growth and current light was the largest.

### Variation in the contributions of natural and artificial light in experiments with combined lighting

In studies with combined lighting, the contribution of natural light to total integrated light at the top of the canopy varied from 24 to 64% (average ± SE = 41.7 ± 1.5%), demonstrating a significant contribution of natural light. Integrated natural light gradient through the canopy (ratio of integrated light at canopy top to bottom) varied from 2.5- to 17-fold, on average 11.7-fold gradient between canopy top to bottom. The gradient in total light (natural plus supplemental) varied from 4.1- to 57-fold (without treatments with intracanopy lighting), on average 26-fold, indicating a much stronger gradient in artificial light, from 8.2- to almost 200-fold, on average 91-fold between canopy top and bottom.

**Figure 3:**
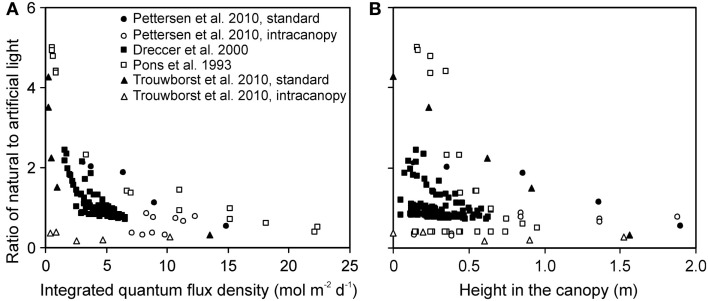
**Ratio of the solar to artificial light in relation to total integrated daily quantum flux density within-canopy (A) and height from the bottom of the canopy (B) in studies conducted in greenhouses with supplemental lighting**. The share of natural and artificial light was determined as described in Material and methods (see Estimation of Changes in Light Profiles for Studies with Combined Natural and Supplemental Lighting). The studies were conducted in the canopies of the herb *Cucumis sativus* (Pettersen et al., 2010; Trouwborst et al., 2010), and grasses *Carex acutiformis* (Pons et al., 1993) and *Triticum aestivum* (Dreccer et al., 2000). In *Cucumis sativus*, the experiments were conducted with standard top lighting and with intracanopy lighting, resulting in greater contribution of supplemental lighting at deeper canopy layers (Pettersen et al., 2010; Trouwborst et al., 2010).

The ratio of natural to artificial light increased with increasing depth in the canopy in all experiments, except for experiments with intracanopy lighting (Figure 3A). The study-to-study variation was large, reflecting differences in stand height, and variations in the share of light at the top of the canopy between artificial and natural light (Figure 3B). The latter variation resulted from differences in integrated natural light (2.2–17.8 mol m^−2^ day^−1^) due to differences of enclosure transmittance and time of the year, and from variations in lamp light output and distance between the luminaire and vegetation.

### Foliage traits in relation to different integrated light estimates

**Figure 4:**
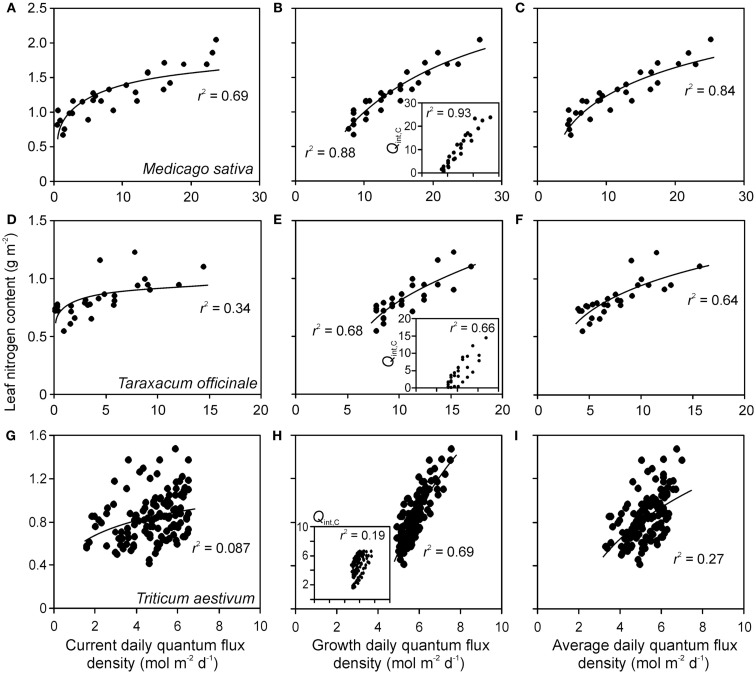
**Sample relationships of leaf nitrogen content per area vs. integrated quantum flux density within the canopy at the time of measurements (current, *Q*_int,C_) (A,D,G), during growth (*Q*_int,G_) (B,E,H) and average of *Q*_int,C_ and *Q*_int,G_ (C,F,I) in herbs *Medicago sativa* (A–C) *Taraxacum officinale* (D–F) (data of Lötscher et al., 2003) and grass *Triticum aestivum***(G–I**)** (**data of Dreccer et al., 2000) grown under completely artificial light in growth chambers (Lötscher et al., 2003) or with supplemental lighting in greenhouses (Dreccer et al., 2000)**. Whenever multiple treatments were available for given species (Table 1), the data were pooled. Insets in **(B,E,H)** demonstrate the correlations between *Q*_int,C_ and *Q*_int,G_. Data were fitted by non-linear regressions in the form of *y* = *a*Ln(*x*) + *b*, that generally provided the highest degree of explained variance (*r*^2^), except for the insets, where *r*^2^ values for linear regressions are provided (all regressions are significant at *P* < 0.001). Details of calculation of *Q*_int,G_ are provided in Material and methods (see Estimation of dynamic changes in light profiles in growth chambers and Estimation of Changes in Light Profiles for Studies with Combined Natural and Supplemental Lighting).

**Figure 5:**
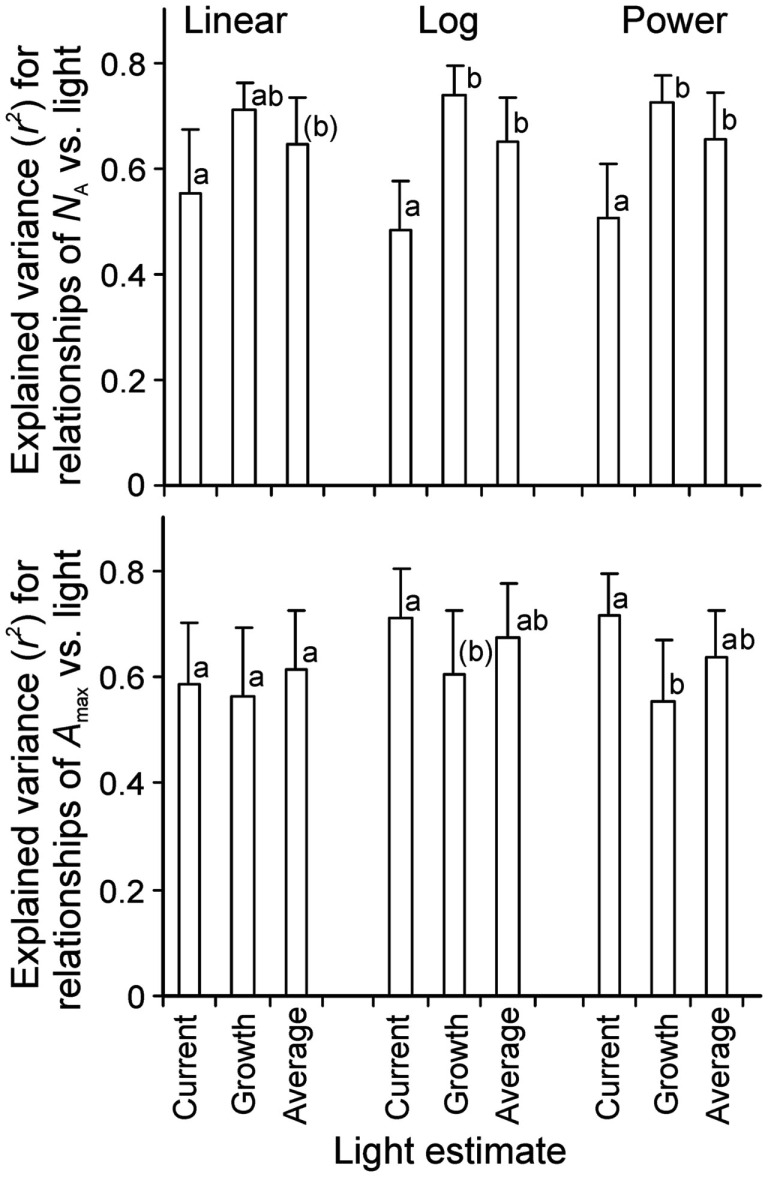
**Average (+SE) explained variance of relationships of nitrogen content pear area (*N*_A_, *n* = 7) and photosynthetic capacity (*A*_max_, *n* = 6) vs. integrated quantum flux density (*Q*_int_) within the canopy**. The integrated light used in the data fitting was either *Q*_int_ at the time of measurements (current, *Q*_int,C_), during growth (*Q*_int,G_), or the average during foliage lifetime until sampling (typically average of *Q*_int,C_ and *Q*_int,G_), and the data were either fitted by linear or non-linear regressions in the form of *y* = *a*Ln(*x*) + *b* (Log) or *y* = *ax^b^* (Power). Figure 4 demonstrates sample relationships of *N*_A_ vs. *Q*_int_ fitted by *y* = *a*Ln(*x*) + *b*. The *r*^2^ data were compared by paired samples *t*-tests and different letters denote statistically significant differences among average *r*^2^ values within given type of regression at *P* < 0.05, except for letters in parentheses that denote differences at *P* < 0.1. Only studies with completely artificial light in growth chambers (Pons et al., 1993; Lötscher et al., 2003) or with partial supply of artificial light in greenhouses (Pons et al., 1993; Xu et al., 1997; Dreccer et al., 2000; Pettersen et al., 2010; Trouwborst et al., 2010) were included in the analyses. The species available in these studies were grasses *Brachypodium pinnatum* (Pons et al., 1993), *Carex acutiformis* (Pons et al., 1993), *Dactylis glomerata* (Lötscher et al., 2003), and herbs *Lycopersicon esculentum* (Xu et al., 1997), *Medicago sativa* (Lötscher et al., 2003), *Taraxacum officinale* (Lötscher et al., 2003), and *Cucumis sativus* (Pettersen et al., 2010; Trouwborst et al., 2010; Table 1 for further information of data sources).

Correlations of *N*_A_ and photosynthetic capacity per area (*A*_max_) with integrated light estimates were explored by linear, logarithmic, and power regressions across studies with artificial and with combined natural and supplemental lighting (Figure 4 for sample relationships with *N*_A_). All three mathematical functions resulted in high degrees of explained variance (*r*^2^, Figure 5). The average explained variance obtained with current integrated light at the time of the measurements (*Q*_int,C_) and with average light through leaf lifetime (*Q*_int,av_) did not generally differ for the three functions (*P* > 0.2), except for the comparison of *r*^2^ for linear and logarithmic regressions with *Q*_int,av_. For this comparison, the average *r*^2^ for logarithmic regressions was marginally higher than that for linear regressions (*P* = 0.06). In contrast, average *r*^2^ for regressions with *Q*_int,G_ differed for all functions (*P* < 0.04), resulting in the following ranking *r*^2^(logarithmic) > *r*^2^(power) > *r*^2^(linear).

Correlations of *N*_A_ with *Q*_int,G_ were generally the strongest, followed by the correlations with *Q*_int,av_ (Figure 4). Statistical differences among average *r*^2^ − *s* for different light estimates were particularly pronounced for fits with logarithmic and power regressions (Figure 5). In contrast, correlations of *A*_max_ with light estimates tended to be the strongest with *Q*_int,C_, followed by *Q*_int,av_ (Figure 5).

### Bias introduced by apparent relative light in foliage trait vs. light relationships

**Figure 6:**
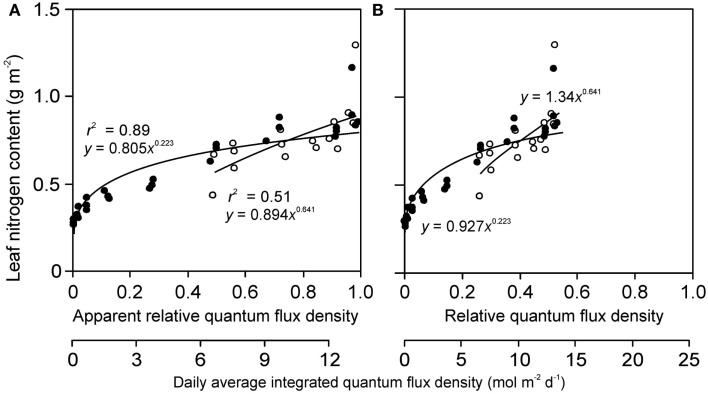
**Sample relationships of leaf nitrogen content per area vs. apparent (A) and actual relative quantum flux density (B) in herb *Lysimachia vulgaris* grown in dense (360 plants m^−2^, filled symbols) and open (40 plants m^−2^, open symbols) stands in a greenhouse (data of Hirose et al., 1988)**. In the original study, the quantum flux density at different locations within the canopy was expressed relative to above canopy values inside the greenhouse. However, this is an apparent estimate of relative quantum flux density (*R*_Q,A_) due to reduction of ambient solar radiation by greenhouse roof and walls (greenhouse wall transmittance κ_Q_). As the actual relative quantum flux density (relative to full sun outdoors), *R*_Q_, is always less than *R*_Q,A_, *R*_Q_ = κ_Q_*R*_Q,A_, the data expressed in relation to true relative light are compressed compared with the data expressed relative to *R*_Q,A_. The absolute light scale is also shown for both panels to underscore this fact. Data were fitted by power regressions that yielded the highest degree of explained variance for the given dataset.

**Figure 7:**
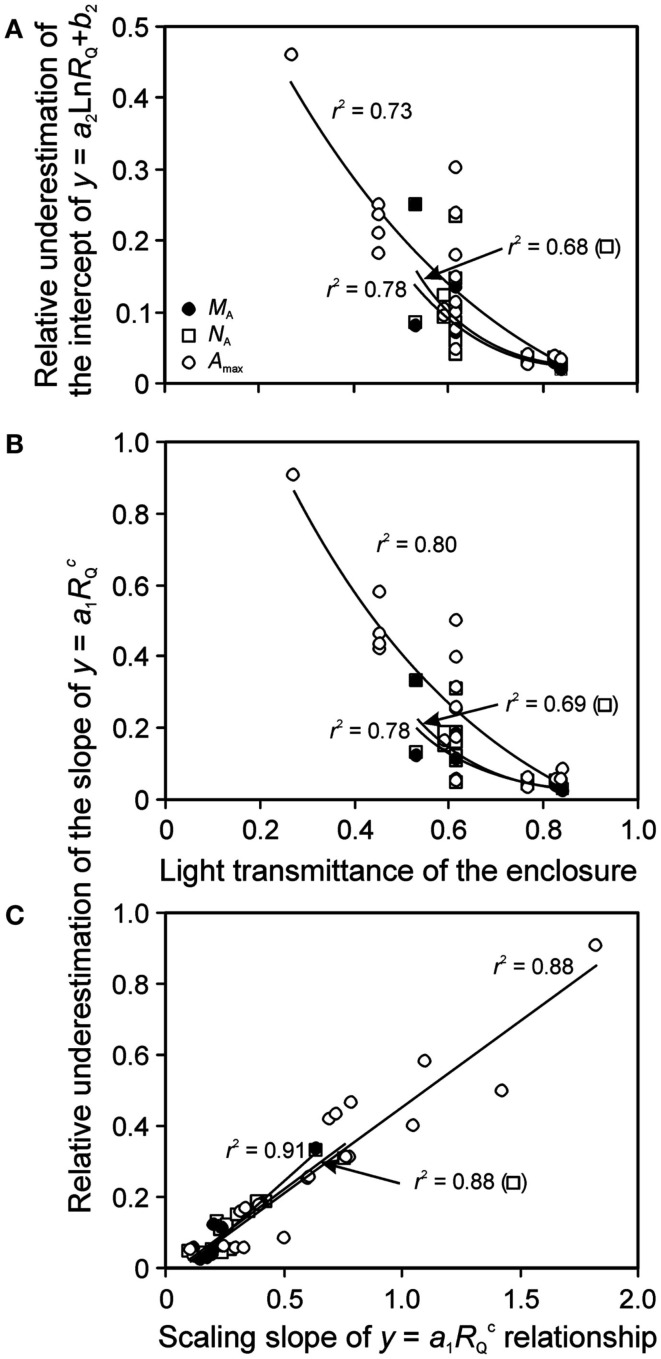
**Bias introduced by lack of consideration of reduction of light by enclosure on the shape of leaf structure, chemistry, and physiology vs. relative light (*R*_Q_) relationships**. Correlations of **(A)** the relative underestimation of the intercept (*b*_2_) of *y* = *a*_2_Ln(*R*_Q_) + *b*_2_ (Eq. 17) and **(B)** the relative underestimation of the slope (*a*_1_) of $y=a_{\text{1}}R_{Q}{\;}^{c}$ (Eq. 16) by using the apparent relative light (*R*_Q,A_) with the light transmittance of the enclosure, and the correlation between the slope (*a*_1_) and scaling exponent **(C)** of $y=a_{\text{1}}R_{Q}{\;}^{c}$ across the studies investigating within-canopy variation in foliage characteristics in greenhouses (Table 1 for the studies included). The relative change is defined as [*p*_j_(*R*_Q_) − *p*_j_(*R*_Q,A_)]/*p*_j_(*R*_Q_), where *p*_j_ refers to a given regression parameter. In addition to leaf nitrogen content per area (*N*_A_) and photosynthetic capacity (*A*_max_), bias in leaf dry mass per unit area (*M*_A_) relationships is also analyzed. The correlations were fitted by non-linear or linear regressions whichever provided the best fit (all regressions are significant at *P* < 0.001). Data sources are provided in Table 3.

Although it is established that plant plastic responses to light environment are driven by the absolute integrated light, *Q*_int_, relative light is often used in acclimation studies. For enclosure studies, this is often the apparent relative light, *R*_Q,A_, i.e., the light intensity relative to that at the top of vegetation in the enclosure rather than light intensity relative to full sunlight (relative to light outside the enclosure, *R*_Q_). The use of *R*_Q,A_ resulted in more extended light range, and shallower leaf trait vs. light relationships (Figure 6 for sample *N*_A_ vs. *R*_Q,A_ and *R*_Q_ relationships). As Eqs 16 and 17 predict, the bias in the regression characteristics scales with the transmittance of the enclosure (κ_Q,av_). The relative change due to use of *R*_Q,A_ (Eq. 15) in the regression intercept of the logarithmic regression for all key traits studied – *M*_A_, *N*_A_, and *A*_max_ – increased with decreasing κ_Q,av_ (Figure 7A). The relative change of the slope of power regression also scaled negatively with κ_Q,av_ (Figure 7B; Table 3 for the changes across different studies and treatments), and positively with the scaling exponent (Figure 7C; Eq. 16) for these traits.

The effect of using *R*_Q,A_ on regression characteristics was similar for all traits, and the relative changes for different traits were generally strongly correlated with each other (*r*^2^ = 0.76 − 0.996), with the exception of weak correlations with the change of the slope of the power function (Eq. 16) for *A*_max_ and *N*_A_ (*r*^2^ = 0.15, *P* = 0.18) and change of the logarithmic function intercept (Eq. 17) for the same traits (*r*^2^ = 0.33, *P* < 0.05). The numerical values of relative changes of given regression characteristics were generally also not significantly different for different traits. Only the difference in relative change in the slope of power regression for *M*_A_ was marginally different from that of *A*_max_ (*P* = 0.10 according to a paired *t*-test).

## Discussion

### Light in artificial environments

As demonstrated in this study, light in artificial environments differs from outdoor light in several important respects. First, in enclosures with natural illumination, maximum light intensity is always less than that outdoors due to light absorption and reflection by enclosure surface (Table 1). Second, in studies with artificial lighting, light gradients in vegetation are not only dependent on canopy characteristics, but are also strongly driven by the distance between light source(s) and vegetation (Figure 1; Poorter et al., 2012). There have been extensive efforts to mimic the spectral composition of light in plant growth applications to be as close as sunlight and some progress has been made by combining different light sources and improving the spectral output of different lamps (Gaastra, 1970; Heathcote et al., 1979; Cathey and Campbell, 1980; Hartmann and Kaufmann, 1989), but still the light spectrum of artificial growth environments is often far from ideal. In particular, achieving red to far red ratios similar to sunlight is problematic (Cumming, 2011), and such modification in light spectral quality can have important implications on key plant structural and physiological traits (Rousseaux et al., 1996; Pons and de Jong-van Berkel, 2004). On the other hand, a meta-analysis demonstrated a surprisingly weak effect of red to far red ratio on leaf dry mass per unit area (Poorter et al., 2009a). As shown in the current study, distance-dependent reduction of light intensity remains a further inherent major limitation in experiments with top lighting. Furthermore, in experiments with combined natural and artificial lighting both the transmittance effects and distance effects require consideration.

### Transmittance of solar irradiance by enclosures

In greenhouse studies, there is surprisingly little information on the average transmittance characteristics in specific experiments. Furthermore, we note that typical measurements of transmittance of greenhouses or environment-controlled enclosures used in plant acclimation studies have been conducted under high solar elevation angles when the solar beams are close to perpendicular to the enclosure surface. As the transmittance decreases with increasing angle of incidence [see Theory: Light in Manipulated Environments (Microcosms, Greenhouses, Growth Chambers)], such point transmittance estimates would significantly underrate the role of enclosure transmittance on daily integrated light. For example, although the transmittance of standard glass is high 0.85–0.9 for perpendicular beam irradiance (Papadakis et al., 2000; Pollet and Pieters, 2000), average glasshouse transmittance is typically on the order of only 0.5–0.6 (Kittas et al., 1999), implying that consideration of enclosure transmittance is of paramount significance.

Multiple factors, including the geometry of the enclosure, weather conditions, latitude, and time of the year can alter the enclosure light transmittance [see Theory: Light in Manipulated Environments (Microcosms, Greenhouses, Growth Chambers)]. Some of these effects are hard to consider *a posteriori*, as many important details such as the exact geometry of the enclosure, reflectance characteristics of the enclosure materials, etc., are typically not reported in original studies. Thus, we derived the average transmittance values of the enclosure, κ_Q,av_, relying on reported optical characteristics of the enclosure material as dependent on the angle of incidence (Kittas et al., 1999; Papadakis et al., 2000; Pollet and Pieters, 2000). Although a simplified approach was used, transmittances derived in our study (Table 1) were within the general range of values reported for greenhouses (Kittas et al., 1999). In addition, the range of available estimates of transmittance in the within-canopy acclimation studies (Table 1) was similar to the range of κ_Q,av_ derived here. Overall, the reported and derived κ_Q,av_ values demonstrate that light was reduced by 15–73%, on average 40–50% in greenhouses, indicating that enclosure transmittance is a major factor determining light availability in the enclosure. As discussed in Section “Enclosures with Natural Lighting” surrounding vegetation and buildings outside the enclosure can further reduce the light availability, and in future studies, we suggest that the effects of such structures on incident light availability should be quantified.

### Within-canopy light profiles in studies with artificial light

The theory predicts that for a completely isotropic light source, the intensity of radiation decreases with the square of the distance (Eq. 6). However, lamps in plant growth studies are generally not isotropic, and their light field depends on lamp and illuminaire (and reflector) geometry. Nevertheless, light field measurements for typical lamps used in plant studies, standardized with respect to quantum flux density relative to the measurement at a fixed distance from illuminaire surface *d*_s_ (0.4 m), yielded a single strong empirical relationship between the distance from the lamp and *Q* (Figure 1, Eq. 12), and this relationship was verified by an independent dataset of Buck-Sorlin et al. (2010; Figure 1). Although a certain departure from the generalized distance vs. intensity relationship is observed for different lamps, the general fit is remarkably good (Figure 1B).

The empirical equation predicted that at 1 m from illuminaire surface, *Q* is reduced by ca. 10-fold, and at 2 m by 45-fold, indicating a major effect of the distance from the lamp on light intensity (Figure 1). This implies that for artificial lighting, changes in vegetation height during stand development are associated with major changes in light available for developing leaves, as a rule, several-fold changes during plant development. Accordingly, the variation of light during foliage development, *Q*_int,G_, is often larger than that in plants growing under natural irradiance (Figure 2). Furthermore, due to distance effect, the overall light gradient from canopy top to bottom is often larger under artificial light, up to 50-fold in our study, than under natural light, typically between 5- and 25-fold (Starzecki, 1975; Hirose and Werger, 1987; Niinemets, 1995; Niinemets and Kull, 1998; Le Roux et al., 1999; Koike et al., 2001), except for field studies with a size hierarchy of individuals (Oberbauer and Strain, 1986; Anten et al., 1998; Hikosaka et al., 1999).

The empirical equation further predicted that the reduction of light intensity with distance, d*Q*/d*d* becomes progressively less with increasing distance *d* from the light source. Thus, the overall sensitivity of integrated plant light to changes in vegetation height (d*Q*_int_/d*h*) depends on the initial distance between the lamp and vegetation. The distances between the light source and chamber floor, and plant heights widely varied across the studies, and this did result in large variation in the range of d*Q*_int_/d*h* across the analyzed stands. Thus, prediction of changes in light availability in growing plants requires not only information on light intensity at the top of vegetation and plant growth rate, but also information on the distance between plants and lamps. Typically, the distance between the plants and light source(s) is kept relatively short to have high *Q* at the top of the vegetation, but this inevitably results in large d*Q*/d*d* and most sensitive changes in *Q* with changing vegetation height.

There are few studies reporting height-dependent light gradients in enclosures. As mentioned in Section “Measurement of Light Fields of Common Lamps Used in Plant Growth Studies,” our results on the magnitude of light gradients agree with Buck-Sorlin et al. (2010). On the other hand, Poorter et al. (2012) reported light gradients of 1.5- to 2.2-fold with distance of up to 1.5 m from the pot level for different growth chambers. This smaller range in Poorter et al. (2012) may seem initially inconsistent with the data in Figure 1B, but without information of the distance of pot to light source, we cannot evaluate the steepness of the light gradient, d*Q*/d*d*, and directly compare our results to Poorter et al. (2012). Although the variation associated with *a posteriori* estimations of lamp height vs. light intensity relationships can be potentially large, our predictions and data of Buck-Sorlin et al. (2010) and Poorter et al. (2012) suggest that major variations in light availability due to changes in the distance between the light sources and vegetation elements are inherent to studies with artificial illumination, especially in studies with fast-growing plants such as herbs and grasses.

### Light profiles in studies with combined lighting

Light intensity in greenhouses with supplemental illumination is the sum of natural and artificial light (Eq. 10). As artificial light intensity depends on the distance from light source, and natural light intensity varies during and among days, the contributions of the two light components change in time and within the canopy (Figure 3). Thus, relative quantum flux density, *R*_Q_, estimated using instantaneous values of *Q* as often used in studies on within-canopy light gradients is bound to vary with all these time-dependent alterations. Therefore, *R*_Q_ based on integrated *Q*, and integrated *Q* itself are more pertinent variables to characterize light environment in studies with combined lighting, and we argue that future studies should be based on these estimates only.

There is an overall large variation among the studies in the share of contributions of artificial and natural light at the top of the canopy, more than an order of magnitude, reflecting differences in time of the year of the study, greenhouse transmittance and light output of artificial light sources. Another key implication of distance-dependent changes of artificial light intensity is that the contribution of different light components varies within the canopy (Figure 3), and it changes due to vegetation growth that alters the distance of canopy elements from artificial light source. In the current study, we have proposed a methodology to separate the contributions of artificial and natural illumination based on retrospective analyses of light intensity vs. distance relationships, measured light gradients within vegetation, enclosure transmittance, and integrated average irradiance above the vegetation. We suggest that in future studies, an effort should be made to measure separately light gradients with natural (e.g., artificial light sources temporarily switched off during daytime) and artificial light (during night).

### Different integrated light estimates for studies on plant acclimation

There is a general consensus that foliage acclimates to integrated average light rather than to maximum instantaneous light intensity in a given environment (Chabot et al., 1979; Niinemets et al., 2004; Poorter et al., 2009a, 2010). As light availability continuously changes in the lower canopy due to vegetation growth, especially in fast-growing canopies, a key question is to what integrated light signal the foliage acclimates. Foliage structural traits, such as *M*_A_, typically are determined early in growing leaves, and after cell wall rigidification, *M*_A_ of mature leaves is relatively invariable (Naidu and DeLucia, 1997a,b; Yamashita et al., 2002; Oguchi et al., 2006), although in younger leaves, *M*_A_ may partly acclimate to altered light climate (Pons and Pearcy, 1994; Yamashita et al., 2002).

In the case of *N*_A_, it is important to consider that it is the product of *M*_A_ and nitrogen content per dry mass (*N*_M_, *N*_A_ = *M*_A_*N*_M_), and within-canopy variation in *N*_A_ can be driven by both of its components. In tree canopies where a new leaf flush is formed almost simultaneously in the beginning of the growing season, and no new leaves are formed during the rest of the growing season, *N*_M_ varies relatively little within the canopy, and thus, *N*_A_ is mainly determined by *M*_A_ and is therefore expected to respond to the same light signal as *M*_A_ (Niinemets, 2007; Niinemets and Anten, 2009; Dewar et al., 2012). In fast-growing canopies with continuous leaf turnover, *N*_M_ can decline in the lower canopy due to induction of leaf senescence that triggers nitrogen resorption (e.g., Niinemets and Anten, 2009), and consequently, *N*_A_ can also respond to light signals later in the ontogeny. Nevertheless, even in herb canopies that have completed the height growth, high *N*_M_ values in the lower canopy can be maintained until leaf senescence (Hikosaka et al., 1994; Hikosaka and Hirose, 2001; Yasumura et al., 2007). This implies that in mature herbaceous canopies where leaf senescence in the lower canopy has not yet been induced, *N*_A_ gradient is also expected to strongly depend on *M*_A_.

As with *N*_A_, foliage photosynthetic capacity per area (*A*_max_) is the product of *M*_A_ and photosynthetic capacity per dry mass. *A*_max_ is strongly correlated with *M*_A_ and *N*_A_ through light gradients (Pons and Anten, 2004; Anten, 2005; Niinemets, 2007; Niinemets and Anten, 2009). Although modifications in *A*_max_ in fully developed leaves are constrained by leaf anatomy, *A*_max_ can adjust to altered light availability as the result of modifications in nitrogen allocation among rate-limiting photosynthetic proteins and light-harvesting pigment-binding complexes and by changes in chloroplast dimensions (Oguchi et al., 2003, 2005, 2006).

This evidence collectively implies that it is important to analyze light environment through canopy development, especially in short-term experiments with fast-growing species in artificial environments where light changes particularly strongly. However, light gradients are typically assessed in mature canopies immediately before foliage sampling (current *Q*_int_, *Q*_int,C_). Here we retrospectively assessed *Q*_int_ during foliage formation (*Q*_int,G_), and during foliage lifetime from development to harvesting (*Q*_int,av_). The explanatory power of the *N*_A_ vs. *Q*_int_ relationships was generally the largest with *Q*_int,G_, followed by *Q*_int,av_ and the weakest relationships were generally observed with current light estimates, especially for non-linear fits (Figures 4 and 5). This agrees with the hypothesis that *N*_A_ is determined by the early light signal.

Overall, poor relationships of *N*_A_ vs. *Q*_int,C_ when multiple treatments are pooled (Table 1 for treatments), and excellent relationships when *Q*_int,G_ is used as the explanatory variable (Figures 4 and 5) further suggest that part of the treatment effects on within-canopy distribution of foliage traits is actually associated with treatment effects on light environment, e.g., on plant to lamp distance due to changes in plant size, rather than treatment effects on the inherent distribution pattern itself. Testing this suggestion requires additional experiments in growth chambers across different treatments explicitly characterizing foliage light environment through leaf lifetime. Nevertheless, outdoor studies have demonstrated analogous strong effects of early light signals (Niinemets et al., 2004; Oguchi et al., 2006), even influences of light availability during bud-set have been demonstrated (Uemura et al., 2000). However, derivation of growth vs. current light estimates for fast-growing canopies in the natural stands in the field may be more complicated because there is often a pronounced variation in age and size of individuals (Anten et al., 1998; Hikosaka et al., 1999; Hikosaka and Hirose, 2001).

In contrast to the relationships with *N*_A_, an opposite pattern was observed with *A*_max_, where the highest explained variances were generally achieved with current, followed by lifetime average and growth light (Figure 5). This suggests that acclimation in photosynthetic capacity can be relatively fast such that current light availability is a stronger determinant of leaf photosynthetic potential than the light availability encountered in the past.

### Bias of using apparent relative light in fitting leaf trait vs. light responses

The slopes of regressions of foliage traits vs. integrated or relative light are often used as estimates of foliage plasticity (Chazdon and Kaufmann, 1993; Nicotra et al., 1997; Niinemets et al., 2001; Portsmuth and Niinemets, 2007). Leaf trait vs. light relationships are also employed in exploring the hypotheses about mechanisms of light acclimation, including the hypotheses about optimality and evolutionary stable strategies (Hirose et al., 1988; Hollinger, 1996; Anten and Hirose, 2001; Anten, 2002). Although *Q*_int_ is the pertinent variable in investigating plant plastic responses to light environment, relative light is still often used. As shown in this study, relative light intensity in enclosures, estimated as the ratio of integrated light inside the vegetation to that at the top of vegetation, *R*_Q,A_ is an apparent estimate of relative light that is always higher than that estimated relative to full sunlight *R*_Q_ (*R*_Q_ = κ_Q,av_*R*_Q,A_). Thus, use of *R*_Q,A_ as the explanatory variable in leaf trait vs. light relationships results in stretched responses (Figure 6), underestimating foliage responsiveness to light. For linear regressions, the relative underestimation of regression slope (Eq. 15) is inversely proportional to κ_Q,av_. In the case of non-linear fits, changes in the regression parameters (slope for the power regression and intercept for the logarithmic regression) scale inversely with κ_Q,av_ (Figure 7), but also depend on the other regression parameters (Eqs 16 and 17). As our study demonstrates, use of *R*_Q,A_ introduces a major bias in the relationships of foliage traits vs. light, and this needs consideration when comparing trait vs. light relationships across studies and in constructing meta-databases on plasticity (e.g., Poorter et al., 2009b, 2010, 2011). Although exact estimation of κ_Q,av_ and regression bias may not be always possible, the important point is that relative light vs. trait relationships estimated indoors and outdoors are not necessarily comparable.

## Conclusion: Acclimation Studies in Artificial Environments

A number of influential pioneering studies on foliage acclimation to within-canopy light gradients have been conducted in artificial environments (e.g., Gutschick and Wiegel, 1988; Hirose et al., 1988; Schieving et al., 1992; Evans, 1993a,b; Pons et al., 1993; Pons and Jordi, 1998; Pons and Anten, 2004), and artificial environments remain an important platform to gain insight into plant acclimation (Table 1, Poorter et al., 2012). However, the major message of the current study is that light intensity and light gradients can importantly differ among artificial and natural environments. In studies conducted with artificial light, the key issue is that the light gradient depends on the distance between light source and vegetation. This is particularly relevant for studies with fast-growing species such as herbs and grasses, most commonly used in growth chamber and greenhouse studies (Table 1). In enclosures illuminated with natural light, reduction in light intensity by enclosure requires consideration when comparing the shapes of the statistical relationships with those in the field. So far, these important aspects have not been given due consideration. We argue that intrinsic differences in light environment between natural and enclosure studies need to be explicitly addressed in future studies and in meta-analyses summarizing information gained in the past, and recommend that in future studies, the integrated absolute light, *Q*_int_, be used as a measure of light availability.

## Conflict of Interest Statement

The authors declare that the research was conducted in the absence of any commercial or financial relationships that could be construed as a potential conflict of interest.

## Abbreviations

*A*_max_, leaf photosynthetic capacity per unit area (μmol m^−2^ s^−1^); *d*, distance from the light source, *r *− *h* (m); *d*_s_, standardized distance from illuminaire surface (taken as 0.4 m in the current study) (m); *f*_dif_, fraction of diffuse light; *g*_1_ – *g*_4_, empirical parameters characterizing distance-dependent reduction in light intensity (Eq. 11); *h*, height relative to chamber floor (m); *h*_0_, top height of vegetation (m); *k*_1_, extinction coefficient for artificial light; *k*_2_, extinction coefficient for natural light; *k*_int_, extinction coefficient for integrated natural light; *L*_c_, cumulative leaf area index (m^2^ m^−2^); *M*_A_, leaf dry mass per unit area (g m^−2^); *N*_A_, leaf nitrogen content per unit area (g m^−2^); *p*_j_, regression parameter *j* (Eq. 15); *Q*, photosynthetically active quantum flux density (μmol m^−2^ s^−1^); *Q*_0_, *Q* incident to the canopy (μmol m^−2^ s^−1^); *Q*_0,L_, *Q* at lamp surface (μmol m^−2^ s^−1^); *Q*_L_, *Q* of artificial light without plants (μmol m^−2^ s^−1^); *Q*_ab_, *Q* incident to enclosure surface (μmol m^−2^ s^−1^); *Q*_en_, *Q* penetrating the enclosure material (μmol m^−2^ s^−1^); *Q*_int_, integrated quantum flux density (mol m^−2^ day^−1^); *Q*_int,0_, *Q*_int_ incident to the canopy (mol m^−2^ day^−1^); *Q*_int,ab_, integrated quantum flux density incident to the enclosure (mol m^−2^ day^−1^); *Q*_int,av_, average *Q*_int_ through leaf lifetime (mol m^−2^ day^−1^); *Q*_int,en_, *Q*_int_ for light penetrating the enclosure material (mol m^−2^ day^−1^); *Q*_int,C_, *Q*_int_ at the time of measurements (mol m^−2^ day^−1^); *Q*_int,G_, *Q*_int_ during growth (mol m^−2^ day^−1^); *Q*_int,L_, integrated quantum flux density for given height in the chamber without plants (mol m^−2^ day^−1^); *r*, distance between the illuminaire surface and chamber floor (m); *R*_Q_, relative quantum flux density, i.e., instantaneous or integrated quantum flux density at given location inside the vegetation relative to quantum flux density above the canopy (full sunlight); *R*_Q,A_, apparent relative quantum flux density, i.e., relative light in an enclosure; *w*_j_, extinction coefficient characterizing reduction of light with depth into the canopy (m^−1^); Δ_P_, photoperiod length (h); κ_Q_, transmittance of enclosure material for photosynthetically active quanta; κ_Q,av_, average κ_Q_ for *Q*_int_ and average *R*_Q_; κ_Q,diff_, κ_Q_ for diffuse light; κ_Q,dir_, κ_Q_ for direct light; χ, photometric to quantum unit conversion factor (lm s μmol^−1^); *υ*, relative change in regression parameter *p*_j_ (Eq. 15); Φ, angle of incidence; Φ, lumen output of a lamp (luminous flux, lm).
